# Exploring the Action Mechanism and Validation of the Key Pathways of *Dendrobium officinale* Throat-clearing Formula for the Treatment of Chronic Pharyngitis Based on Network Pharmacology

**DOI:** 10.2174/0113862073261351231005111817

**Published:** 2024-02-09

**Authors:** Xi Fang, Xiao-Feng Jiang, Yi-Piao Zhang, Cheng-Liang Zhou, Ying-Jie Dong, Bo- Li, Gui-Yuan Lv, Su-Hong Chen

**Affiliations:** 1 Collaborative Innovation Center of Yangtze River Delta Region Green Pharmaceuticals, Zhejiang University of Technology, No. 18, Chaowang Road, Gangshu District, Hangzhou, Zhejiang, 310014, China;; 2 Zhejiang Provincial Key Laboratory of TCM for Innovative R & D and Digital Intelligent Manufacturing of TCM Great Health Products, No. 999 Changhong East Street, Huzhou City, Zhejiang, 310023, China;; 3 College of Pharmaceutical Science, No. 548, Binwen Road, Binjiang District, Zhejiang Chinese Medical University, Hangzhou, Zhejiang, 310053, China

**Keywords:** *Dendrobium officinale*, chronic pharyngitis, throat-clearing, network pharmacology, TLR4/PI3K/Akt/NF-κB, CP process

## Abstract

**Aim:**

This study investigated the molecular action mechanism of a compound herb, also known as the *Dendrobium officinale* throat-clearing formula (QYF), by using network pharmacology and animal experimental validation methods to treat chronic pharyngitis (CP).

**Methods:**

The active ingredients and disease targets of QYF were determined by searching the Batman-TCM and GeneCards databases. Subsequently, the drug-active ingredient-target and protein-protein interaction networks were constructed, and the core targets were obtained through network topology. The Metascape database was screened, and the core targets were enriched with Gene Ontology and the Kyoto Encyclopedia of Genes and Genomes.

**Results:**

In total, 1403 and 241 potential targets for drugs and diseases, respectively, and 81 intersecting targets were yielded. The core targets included TNF, IL-6, and IL-1β, and the core pathways included PI3K-Akt. The QYF treatment group exhibited effectively improved general signs, enhanced anti-inflammatory ability *in vitro*, reduced serum and tissue expressions of TNF-α, IL-6, and IL-1β inflammatory factors, and decreased blood LPS levels and Myd88, TLR4, PI3K, Akt, and NF-κB p65 protein expression in the tissues.

**Conclusion:**

QYF could inhibit LPS production, which regulated the expression of the TLR4/PI3K/Akt/NF-κB signaling pathway to suppress the expression of the related inflammatory factors (*i.e*., TNF-α, IL-6, and IL-1β), thereby alleviating the CP process.

## 1 INTRODUCTION

Chronic pharyngitis (CP) involves chronic inflammation of the mucosa, submucosa, and lymphatic tissues in the pharynx, a part of the upper respiratory tract [1]. This CP characteristic is the cause of ongoing discomfort and irritation in the throat and requires continuous management and treatment [2]. Clinical symptoms of CP are mainly redness, pain, dryness of the throat, discomfort when swallowing, and dry cough with little sputum, and it is mostly accompanied by fever, other symptoms of upper respiratory tract infection, loss of appetite, and other systemic symptoms [3]. More than 20% of adults globally have CP, and in China, CP patients account for one-third of total visits to physicians for issues with the ear, nose, and throat (ENT) [4, 5]. Strong evidence indicates that the inflammatory response is a vital player in the pathogenesis of non-infectious pharyngitis. Repeated and acute inflammatory injury may cause chronic ENT disease and increase the susceptibility of the nasopharyngeal mucosa to carcinogenesis [6, 7]. With the increasing pressure of life, modern people often adopt hot pot, barbecue, alcohol consumption, and bad lifestyle habits to relieve anxiety [8]. In people consuming spicy food over the long term, drinking stimulates the throat, causing CP. A high percentage of CP patients consume spicy food and have drinking habits. This poor lifestyle is inseparable from CP onset [9].

Presently, anti-infective therapies are considered among the main methods for CP treatment [10-14]. Although these therapies can improve the condition, they are associated with several side effects, symptom recurrence, and late drug resistance and can only relieve short-term emergencies. The clinical management of non-infectious pharyngitis remains controversial [15]. Traditional Chinese Medicine (TCM) is a crucial part of complementary and alternative medicine. CP treatment using TCM theories is advantageous as it offers less toxic side effects and mild and long-lasting curative effects [16-18].

*Dendrobium officinale* Kimura et Migo is a herb (dry stem) belonging to the Orchidaceae family. It benefits the stomach, nourishes yin, and clears heat. Laboratory studies have revealed that crude polysaccharides and non-polysaccharides of *Dendrobium officinale* have anti-CP properties [15]. However, whether the *Dendrobium officinale* throat-clearing formula (QYF), composed of *Dendrobium officinale* as the monarch drug and in a reasonable combination, has the same good pharmacological effect on CP induced in humans by consuming spicy food and alcoholic beverages in excess and whether QYF reduces the release of inflammatory factors through the inflammatory signaling pathways warrant further explorations. No systematic study has yet investigated this topic because of the complexity of the chemical composition of most TCMs. QYF is associated with similar problems, such as unclear active ingredients and unknown therapeutic targets. The holistic and systematic nature of pharmacology and the focus on drug-drug interactions match the basic TCM characteristics and are also consistent with the understanding of the disease’s nature in TCM [19-22]. Moreover, using network pharmacology for studying Chinese medicine is beneficial for its modernization. Therefore, network pharmacology can be applied to analyze the active ingredients and the therapeutic targets of QYF.

This study adopted a network pharmacology approach to identify the active ingredients, key targets, and relevant signaling pathways of QYF for CP treatment. A CP model induced through excessive consumption of a mixture of chili water and alcohol was innovatively developed to assess the QYF pharmacodynamics and validate network pharmacology-predicted molecular targets and signaling pathways. The study results will facilitate the further investigation of potential action mechanisms of QYF in CP treatment.

## 2 MATERIALS AND METHODS

### 2.1 Materials

Qing Hou Li Yan Ke Li (MYSN, F220508) were purchased from Anhui Guilong Pharmaceutical Co., Ltd (Anhui, China). TNF-α Elisa Kit (202211), IL-1β Elisa Kit (A301B30226), and IL-6 Elisa Kit (202211) were purchased from Hangzhou Unitech Biotechnology Co., Ltd (Hangzhou, China). Rat LPS Elisa Kit (202304) was purchased from Shanghai Yuancheng Biotechnology Center (Shanghai, China). NF-κB p65 antibody (20k7668) and TLR4 antibody (16c5074) were purchased from Jiangsu ProTech Biological Research Center Co., Ltd (Jiangsu, China). Myd88 antibody (10022637), AKT antibody (00114013), PI3K antibody (00105490), TNF-α antibody (00077108), IL-1β antibody (00103356), and IL-6 antibody (10017944) were purchased from Proteintech, U.S.A. *Dendrobium officinale* (220801) was purchased from Zhejiang Senyu Holding Group Co., Ltd. (Zhejiang, China). *Chrysanthemi Flos* (220800), *Polygonatum cyrtonema* (22080), *Platycodon grandiflorum* (220301), *Glycyrrhiza uralensis* (220601), and *Mume Fructus* (220601) were purchased from Zhejiang Chinese Medical University.

### 2.2 Animals and Experiment Design

Fifty SPF-grade SD male rats were selected and provided by Shanghai Slake Experimental Animal Co., Ltd, with license number SCXK (Zhejiang) 2022-0004. Feeding conditions employed were room temperature 22-26°C, relative humidity 50~70%, alternating light and dark for 12 hours, maintaining good ventilation, no large noise interference, and free intake of water. All experiments were performed in accordance with the Regulation of Experiment Animal Administration issued by the Ministry of Science and Technology of the People’s Republic of China. The experiment was approved by the ethics committee of Zhejiang University of Technology (Approval no. 20220004004088).

As shown in Fig. (**1**), after one week of acclimation, fifty healthy male SD rats were randomly divided into a blank control group (10 rats) and a modeling group (40 rats) according to body mass. The rats in the modeling group were given a mixture of alcohol-chili pepper liquid (1:1) by gavage at 0.01 mL/g body mass daily and sprayed into the rats' throats for stimulation. On the first day, the rats were given a mixture of 15-degree alcohol and 0.4 g/mL of chili pepper liquid once a day, and then the degree of alcohol increased by 10 degrees. The mass concentration of chili pepper liquid increased by 0.2 g/mL every day, and the mixture of 55-degree alcohol and 1 g/mL of chili pepper liquid was sprayed into the pharynx of the model rats with a throat sprayer (1 time/d, 3 times/each). The normal group was sprayed with the same amount of ultrapure water as the control. At the same time, the rats in the modeling group drank 15 degrees alcohol with 0.4 g/mL of alcohol-pepper solution (1:1), and the normal group drank ultrapure water. After 3 weeks of modeling, blood was collected from the orbits of rats after 12 hours of fasting without water to measure the whole blood routine, and the model group was randomly divided into 4 groups.

The modeled groups were randomly divided into four groups based on leukocyte count and body weight, including the model control group, QYF-H group (0.25 g/kg, QYF of high dose), QYF-L group (0.125 g/kg, QYF of low dose), and MYSN group (3 g/kg), with 10 rats in each group. The rats were administered by gavage daily for 6 weeks while modeling, except for the blank control and model groups. The normal control and model groups were given the same volume of ultrapure water for 6 weeks. The animals were euthanized after completion of the experiment, and the rats were anesthetized by intraperitoneal injection of 2% sodium pentobarbital at a dose of 0.3 mL/100 g, which was proven to be applicable by literature review and previous experimental experience.

### 2.3 Screening of Potentially Active Compounds in QYF

The information on the main active compounds of QYF was retrieved through the Traditional Chinese Medicine Systems Pharmacology Database and Analysis Platform (TCMSP) and a Bioinformatics Analysis Tool for Molecular Mechanism of Traditional Chinese Medicine database. The information on the main active compounds of QYF was retrieved from the database of Traditional Chinese Medicine (Batman-TCM). The chemical composition structure of *Dendrobium officinale* was referred to in the literature. The active ingredients of *Dendrobium officinale* were obtained in the TCMSP database with the oral bioavailability (OB) option set to ≥ 30% and the drug-likeness (DL) option set to ≥ 0.18. BATMAN-TCM was used to search *Chrysanthemi Flos*, *Polygonatum cyrtonema*, *Platycodon grandiflorum*, *Glycyrrhiza uralensis* and *Mume Fructus*, and score cutoff = 20 and adjusted *P*-value = 0.05 were used as the screening criteria of active ingredients and action targets to obtain the active ingredients and their corresponding targets.

### 2.4 QYF Target Acquisition

The target protein corresponding to the active ingredient of *Dendrobium officinale* was selected in TCMSP and entered into the Uniprot database with the species option set to “*Homo sapiens*” to obtain the corresponding target gene.

### 2.5 CP-related Target Collection

The GeneCards database was searched for “Chronic pharyngitis” targets, and a score of ≥10 was used as the screening criteria for CP-related targets. The active compound targets of the screened core drugs and the CP targets were entered into Venny 2.1 to obtain the intersection of the drug pair and the CP targets, *i.e.,* the common targets.

### 2.6 Establishment of the Network

The active ingredients and common targets were entered into Cytoscape 3.9.1 software, and a network diagram of “active ingredients-common targets” was constructed. The core active ingredient of the core drug was screened according to degree by the Network Analyzer function. The drug and disease common target was entered in the STRING 11.0b database, and the protein type was set as “*Homo sapiens*”. Afterward, the protein interaction relationship was obtained, and the protein interaction information was imported into Cytoscape 3.9.1 to get protein-protein interaction (PPI). The node size was reflected by the degree value.

### 2.7 Enrichment Analysis

We imported the common targets of core drugs and CP in the Metascape database, predicted the GO function enrichment analysis of the biological process (BP), molecular function (MF), and cellular component (CC) of core drugs for the treatment of CP, and selected the top 10 entries with the smallest *P*-value for each result. Then, we performed the KEGG pathway enrichment analysis, selected the top 30 entries with the smallest P-value for each result, and made a bubble chart of the enrichment analysis on the microbiology letter website.

### 2.8 Gross Pharyngeal View and Routine Blood Tests

The rat pharynx was observed by endoscopy and scored with the scoring criteria, as mentioned in Table **1**.

According to the method described previously [23, 24], blood was obtained from the fundus venous plexus for 12 h without food or water, and 1 mL was placed in an EDTA-coated blood collection tube. Whole blood WBC, NEUT, LYMPH, and MONO counts were measured using a blood system analyzer.

### 2.9 Foot Swelling and Ear Swelling Experiments in Rats

As previously described [25], 1 hour after the final administration, freshly prepared 1% carrageenan gum 0.1 mL was injected into the toe of the right foot of each group, except for the normal control group, which was injected with an equal amount of saline. The rats' toe thickness of the right foot was measured with vernier calipers before and 1, 2, 4 and 6 h after modeling.

Swelling degree (%) = (average thickness after molding - average thickness of foundation) / average thickness of foundation × 100%.

As previously described [26], 1 h after the final administration, 20 μL of xylene was evenly applied to both sides of the right ear of each rat except for the normal group, and the left ear was used as the normal control, while the right ear of the rats in the normal control group was applied with an equal amount of saline. After 0.5 h of xylene application, the rats were anesthetized, and the ear temperature of the right ear was measured with a thermometer. Then, both ears were cut off, and the ear pieces were punched in the same area of both ears with a 7 mm diameter punch and weighed. The difference in weight between the two ears was used as the ear gallery's swelling value to calculate the ear's swelling rate.

Auricular swelling rate (%) = Auricular swelling value/weight of control earpiece × 100%.

### 2.10 Measurement of Inflammatory Markers and Endotoxins

Consistent with the literature approach [27], the rats were weighed after 12 h of fasting without water before the final administration. Then, 2% pentobarbital sodium was injected intraperitoneally to anesthetize the rats. The blood was collected from the abdominal aorta and centrifuged at 3000 r/min for 10 min in an EP tube, and the serum was frozen at -80°C for storage. The levels of IL-1β, IL-6, TNF-α, and LPS in serum were measured according to the kit instructions.

### 2.11 Histological Evaluation and Immunohistochemistry (IHC)

The pharyngeal samples were fixed in 10% formalin solution for 72 hours and then dehydrated [28]. The samples were then embedded in paraffin, cut into 4-μm-thick sections, stained with hematoxylin-eosin (H&E), and observed under a microscope (OLYMPUS BX43, Japan) [29]. Expression and localization of Myd88, TLR4, AKT, PI3K, NF-κB p65, IL-1β, IL-6, and TNF-α in the throat were determined by immunohistochemical staining. Tissue sections were incubated with primary antibodies (1:100, dilution), and then HRP-conjugated goat was added to anti-rabbit IgG. DAB staining showed signals and hematoxylin-stained cell nuclei. Finally, the results of protein levels were assessed by semi-quantitative analysis using Image Pro Plus software.

### 2.12 Statistical Processing

The biological assay results were presented as mean standard deviation. One-way analysis of variance was performed to analyze significant differences in measured traits by using SPSS statistical software. The LSD t-test was applied only when the hypothesis of homogeneity of variance was satisfied. Otherwise, the Dunnet t-test was performed. All the results were considered statistically significant at *p* <0.05.

## 3 RESULTS

### 3.1 Screening of Active Ingredients of QYF

The active constituents of *Dendrobium officinale* were obtained by searching the structure of *Dendrobium officinale* from the literature, setting the oral bioavailability (OB) option to ≥ 30% and the drug-like property (DL) option to ≥ 0.18 in the TCMSP database, and entering them into the Uniprot database with the species, as mentioned in Table **2**. The target genes were obtained by setting the option “*Homo sapiens*”.

The BATMAN-TCM bioinformatics analysis tool for the molecular mechanism of traditional Chinese medicine was used to search for *Chrysanthemi Flos*, *Polygonatum cyrtonema*, *Platycodon grandiflorum*, *Glycyrrhiza uralensis* and *Mume Fructus*, and the score cutoff = 20 and adjusted *P*-value = 0.05 were used as the screening criteria for active ingredients and targets of action. The active ingredients were obtained, as shown in Table **3**.

### 3.2 Prediction of Common Target Between QYF and CP

The GeneCards database was searched for “CP”, and a score ≥ 10 was used as the screening criteria for CP-related targets. Using the Venny website, the 1403 drug candidates were intersected with 241 targets for CP, and 81 targets for CP were obtained from core drugs. The results are shown in Fig. (**2A**) and Table **4**.

### 3.3 Building a Network

Cytoscape 3.9.1 software was applied to construct the “active ingredient-target-drug” network of core drugs for treating CP, which contained 167 nodes and 314 edges, as shown in Fig. (**2B**). The 10 compounds with the most connected targets in order of degree were chrysanthenone, 3-Methyl-6,7,8-Trihydropyrrolo [1,2-a]Pyrimidine-2-One, thymol, betulin, tetrahydropalmatine, hydroginkgolinic acid, caryophyllene, alpha-pinene, beta-pinene, and camphene.

The common targets of the core drug and CP were imported into the STRING database, the species was limited to humans, the protein interaction information was obtained, and the results were saved in tsv format and imported into Cytoscape 3.9.1 software to plot the PPI network related to drug treatment of CP, as shown in Fig. (**2C**). The top 10 targets, according to the degree of their correlation, were TNF, IL-6, IL-1β, AKT1, TP53, STAT3, INS, TLR4, IL-10, and EGFR.

### 3.4 Functional Enrichment Analysis of Target Genes

Biological process(BP)enrichment analysis results are shown in Fig. (**3A**), mainly including positive regulation of protein phosphorylation, positive regulation of phosphorylation, positive regulation of transferase activity, regulation of kinase activity, positive regulation of kinase activity, regulation of protein kinase activity, positive regulation of protein kinase activity, regulation of MAPK cascade, positive regulation of MAPK cascade, and regulation of protein serine/threonine kinase activity.

Molecular function (MF) enrichment analysis results are shown in Fig (**3B**), mainly including signaling receptor activator activity, signaling receptor regulator activity, receptor-ligand activity, cytokine activity, cytokine receptor binding, growth factor activity, growth factor receptor binding, phosphatase binding, protein phosphatase binding, and chromatin binding.

The results of the CC enrichment analysis are shown in Fig. (**3C**). The key active ingredients mainly acted on the vesicle lumen, cytoplasmic vesicle lumen, secretory granule lumen, lytic vacuole, lysosome, azurophil granule lumen, vacuolar lumen, primary lysosome, azurophil granule, and membrane raft.

### 3.5 Core Signaling Pathway Screening

The KEGG signaling pathway enrichment analysis of the potential targets of drug action is shown in Fig. (**3D**). The results showed that the drug action targets were mainly distributed in multiple signaling pathways, such as the TNF signaling pathway, human cytomegalovirus infection, PI3K-Akt signaling pathway, and JAK-STAT signaling pathway, which indicated that QYF could regulate CP through multiple pathways.

### 3.6 Effect of QYF on Blood Routine in CP Rats

Previous studies have shown that using an ammonia-induced CP model in rats resulted in a significant increase in blood WBC, NEUT, LYMPH and MONO counts, pharyngeal mucosal congestion, and swelling [32]. In this study, after 6 weeks of administration, as shown in Fig. (**4A-D**), whole blood WBC and LYMPH counts were significantly lower in the QYF-H group (*P*< 0.01). There was a trend of lower NEUT and MONO counts, but there was no significant difference. Whole blood WBC, LYMPH and NEUT counts were significantly lower in the QYF-L group (*P*< 0.01,0.05), and MONO counts tended to decrease (*P>* 0.05).

### 3.7 Effect of QYF on Foot Swelling and Ear Swelling in Rats

The effect of QYF on foot swelling in rats with CP is shown in Fig. (**5**). After 1 h of injection, the toe swelling of rats in the model control group was significantly higher compared to the normal control group (*P*< 0.01), and the toe swelling of rats in the QYF-H and QYF-L groups was significantly lower compared to the model control group (*P*< 0.01). Notably, the toe swelling of rats in each group also recovered to different degrees 6 h after injection. The rats in the normal control group recovered to near normal levels, and the model control rats recovered more slowly compared to the rats in the normal control group, and the toe swelling of the rats in the QYF-H and QYF-L groups was significantly reduced (*P*< 0.01, 0.05). The above-mentioned findings suggested that QYF-H and QYF-L groups can reduce the foot swelling caused by carrageenan in model rats.

Xylene-induced ear edema is a simple and classical model for inflammation studies and is widely used to assess the anti-inflammatory activity of substances [33]. The results showed that the ear temperature and auricular swelling rate of the model control rats with swollen ears were significantly increased compared to the normal control group (*P*< 0.01, 0.05). After treatment with QYF-H and QYF-L, ear temperature and auricular swelling rate were significantly lower in rats compared to the model control group (*P*< 0.01, 0.05). These findings indicate that QYF-H and QYF-L could improve the ear swelling induced by xylene in rats.

### 3.8 General Health Status of Animals in the Treatment Group

**Fig. (6):**
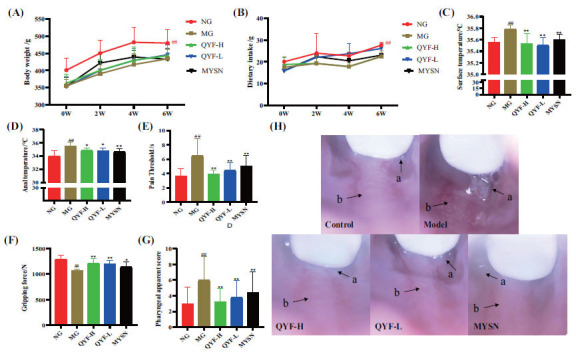
Effect of QYF on the general signs of CP rats. The effects of QYF on the body weight (**A**), feeding (**B**), surface temperature (**C**), anal temperature (**D**), pain threshold (**E**), and gripping strength (**F**) in CP rats. The effect of QYF on the pharyngeal apparent score (**G**) and pharyngeal bulk (**H**) in CP rats after 6 weeks of administration. (**a**) Saliva production. (**b**) Erythema of the pharynx. When compared with the normal control group, ^#^*P* < 0.01, ^#^*P* < 0.05; when compared with the model control group, **P < 0.01, *P < 0.05.

Three weeks after modeling, these animals began to show symptoms, such as yellowing of the fur, frequent scratching and rubbing, irritability, significant weight loss, reduced food intake, significant congestion and swelling of the throat, dilated and red capillaries, significantly increased oral secretions, and occasional bleeding. However, after 6 weeks of QYF treatment, the overall health status of the animals was improved. As shown in Fig. (**6A**-**F**), compared with the model group, QYF-H and QYF-L groups rats showed increased body weight and food intake, significantly lower surface temperature, anal temperature and pain threshold (*P*< 0.01, 0.05), and significantly increased grip strength (*P*< 0.01). Moreover, it was observed by the further pharyngeal gross view that the pharyngeal view status of the treatment group was significantly improved, and pharyngeal scores were significantly lower (*P*< 0.01) compared to the model group after QYF-H and QYF-L groups intervention (Fig. **6G** and **H**).

### 3.9 Observation of the Histology of the Pharynx

Histopathological analysis of the rat pharynx is shown in Fig. (**7**). After H&E staining, the pharyngeal tissue sections of rats in each group were observed under the microscope. In the normal control group, the pharyngeal mucosal vesicles were abundant and closely arranged, the pharyngeal tissue was demarcated in all layers, the submucosa was loose connective tissue, the glands were not hyperplastic and atrophic, and the tissue was not congested, edematous, and fibrous tissue hyperplasia was observed.

Compared with the normal control group, the pharyngeal tissue of the model control rats was infiltrated by inflammatory cells; in the mucosal epithelium, peg-like proliferation appeared, different degrees of glandular disorder were observed, and epithelial cells were shed. In the rats of QYF groups, there was no obvious hyperplasia of pharyngeal mucosal epithelial peg-like protrusions, vascular congestion in the lamina propria improved, inflammatory cell infiltration decreased, along with glandular secretion proliferation, hyperplasia, and mucus secretion, and no fibrous hyperplasia was seen. The above suggests that the QYF can improve the pathological damage of the pharynx caused by CP in rats.

### 3.10 Effect of QYF on Inflammatory Cytokine Levels and Endotoxin in CP Rats

The levels of IL-6, IL-1β, TNF-α, and LPS in rat serum were measured by Elisa kits. As shown in Fig. (**8A**-**D**), compared with normal controls, the release of IL-6, IL-1β, TNF-α, and LPS were significantly increased in the model group (*P*< 0.01, 0.05). Hence, it was demonstrated that QYF-H and QYF-L could significantly decrease the levels of IL-6, IL-1β, and LPS (*P*< 0.01, 0.05), QYF-L could significantly reduce the level of TNF-α (*P*> 0.05), and QYF-H showed a decreasing trend, but no significant difference.

Furthermore, IHC also showed that the expression of IL-1β, IL-6, and TNF-α was significantly increased in the pharyngeal tissues of the model control rats compared to the normal control group (*P*< 0.05). The expression of IL-1β, IL-6, and TNF-α was significantly decreased in the QYF-H and QYF-L group compared to the model control group (*P*< 0.05), and the results are shown in Fig. (**9A**-**F**). These results suggested that QYF-H and QYF-L groups can effectively inhibit the inflammatory response and endotoxin LPS production in CP rats.

### 3.11 Effects on TLR4/PI3K/Akt/NF-κB Signaling Pathway in Rat Pharynx

Among the signaling pathways in which network pharmacology predicts target action, the PI3K/Akt signaling pathway is able to regulate autophagy, which, in turn, regulates inflammation and fibrosis [34, 35]. It has been found that LPS activates PI3K through TLR4 and leads to AKT activation, PI3K promotes NF-κB p65 transcriptional activity and TNF-α production, while inhibition of PI3K leads to a decrease in TNF-α [36]. Therefore, we evaluated the ability of QYF to regulate the TLR4/PI3K/Akt/NF-κB signaling pathway.

The expressions of TLR4, Myd88, AKT, PI3K, and NF-κB p65 proteins in rat pharyngeal tissues were determined by IHC. As shown in Fig. (**10A**-**J**), the levels of TLR4, Myd88, AKT, PI3K, and NF-κB p65 proteins were significantly upregulated in the CP model rats compared to the normal controls (*P*< 0.01). However, the protein levels of TLR4, Myd88, AKT, PI3K, and NF-κB p65 were all decreased after QYF-H and QYF-L intervention (*P*< 0.01, 0.05). These results suggested that QYF-H and QYF-L can inhibit the abnormal activation of the TLR4/PI3K/Akt/NF-κB pathway in rats with CP.

## 4 DISCUSSION

CP is a diffuse inflammation of the mucosa, submucosa, and lymphoid tissues of the pharynx [37]. A high percentage of CP patients consume a spicy diet, indicating that CP incidence can further increase as people's lifestyle changes. Drugs must be developed for the treatment of CP caused by the consumption of spicy foods and alcoholic beverages in excess. Inflammation-targeting therapeutic interventions are crucial for protecting and treating non-infectious CP. Chinese medicine has recently achieved good results in CP treatment [38, 39]. QYF is composed of six herbs, namely *D. officinale*, *Chrysanthemi Flos*, *Polygonatum cyrtonema*, *Platycodon grandiflorum*, *Glycyrrhiza uralensis*, and *Mume fructus*. These herbs exert the effects of nourishing the Yin, moistening the lungs, clearing heat, and improving the throat.

In this study, it was found that after intervention with QYF-H or QYF-L, the treated rats exhibited an increase in body weight and food intake, as well as improvement in the general signs (*i.e*., facial temperature, grip strength, anal temperature, and pain threshold). More importantly, the pharyngeal scores of the treated group significantly reduced after the QYF-H or QYF-L intervention, (*P* < 0.01), indicating that QYF-H and QYF-L had a therapeutic effect on CP rats. Results from the literature show that the inflammatory process is often accompanied by an increase in body temperature, which is consistent with the findings of increased facial and anal temperatures in our model rats during the experimental process, suggesting that QYF has an inhibitory effect on the increase in inflammatory body temperature caused by overconsumption of spicy food and alcoholic beverages [40]. However, the action mechanism of QYF warrants further exploration. Network pharmacology is consistent with the holistic view of TCM, such as discriminatory treatment and treating different diseases together [41]. Therefore, this network pharmacology-based study could reveal the action mechanism of QYF for CP treatment.

In this study, network pharmacology was combined with an experimentally validated approach to investigate the therapeutic action mechanisms of QYF. The network pharmacology prediction results revealed the presence of 81 targets for QYF core drug therapy for CP. The 10 compounds with the most connected targets in the order of degree were chrysanthenone, 3-methyl-6,7,8-trihydropyrrolo [1,2-a] pyramid ine-2-one, thymol betulin, tetrahydropalmatine, hydroginkgolinic acid, caryophyllene, alpha-pinene, beta-pinene, and camphene. Literature research revealed that among the active ingredients obtained through screening, thymol [42], betulinol [43], jensoethol [44], fenugreek ethereal [45, 46], and gastrodene exhibited certain anti-inflammatory, antibacterial, and restoring autophagic activities. According to the PPI network diagram, the top 10 core targets were TNF, IL-6, IL-1β, AKT1, TP53, STAT3, INS, TLR4, IL-10, and EGRF.

The foot and ear swelling and assays are useful tools for the *in vitro* anti-inflammatory evaluation of novel anti-inflammatory drugs [47-49]. The rats were injected with carrageenan gum at the toe in the foot swelling assay, while xylene was applied to both sides of the right ear in the ear swelling assay. GLY had a significant anti-inflammatory effect at 2–6 h in a carrageenan-induced foot edema rat model after the QYF-H and QYF-L combined intervention. In the xylene-treated rats, ear temperature was significantly lower, and the swelling was significantly lower in the QYF-H- and QYF-L-treated group relative to that in the model group. Thus, these findings suggest that the QYF-H and QYF-L combination exerts some anti-inflammatory effects.

CP occurrence and development are closely related to various inflammatory factors [50-52]. A body subjected to external stimuli releases various inflammatory factors in a short period, including IL-6, IL-1β, and TNF⁃α, which damages human tissues and organs [50, 53-55]. TNF⁃α is a T-lymphocyte-secreted cytokine. It is both an initiator of the inflammatory mediator cascade and an effector that synergistically induces other inflammatory factors to act together and damage tissue cells. It has various biological activities [56-58]. IL-1β is a typical pro-inflammatory factor produced by mononuclear macrophages [59-61], and IL-6 is secreted by fibroblasts and activated T cells, which can induce the synthesis and secretion of inflammatory response-associated reactive proteins, and its continued production can cause various autoimmune diseases [62-64]. Our results revealed that, in the treatment group, QYF-H could significantly downregulate the serum TNF-α and IL-1β expression (*P* < 0.05), and QYF-L could significantly downregulate the serum TNF-α, IL-1β, and IL-6 expression (*P* < 0.05). The IHC results indicated that QYF-H and QYF-L together could effectively inhibit TNF-α, IL-1β, and IL-6 levels in the rat pharynx.

**Fig. (11):**
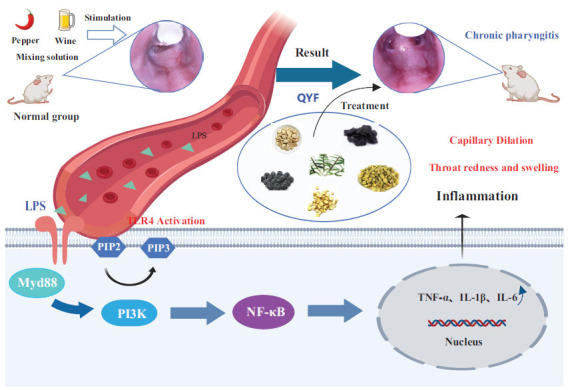
The action mechanism of the TLR4/PI3K/Akt/NF-κB signaling pathway in CP rats. *“Created with MedPeer (medpeer.cn)”.ID: 1b8940bmv4cqntcpv81698152183*.

The enrichment analysis of potential drug targets in the Kyoto Encyclopedia of Genes and Genomes (KEGG) signaling pathway revealed that the drug targets are mainly located in the TNF signaling pathway, human cytomegalovirus infection, PI3K-Akt signaling pathway, JAK-STAT signaling pathway, and other signaling pathways. LPS is a unique outer wall component of gram-negative bacteria, a lipid and polysaccharide complex that is an extremely potent stimulant of the inflammatory response [65, 66]. Toll-like receptor 4 (TLR4) belongs to a TLR family that recognizes relevant molecules on the pathogen’s surface, inducing the activation of downstream signaling pathways, which, in turn, activate the expression of inflammatory factors and trigger inflammatory responses [67]. Myd88 is a typical adaptor of downstream inflammatory signaling pathways of TLR and IL-1 receptor families [68]. Capsaicin and alcohol intake increase serum LPS levels, which bind to TLR4 through Myd88 to activate the downstream inflammatory signaling and mediate inflammatory cytokine production by macrophages [69]. PI3K is a kinase involved in several crucial cellular pathways, and TLR4-mediated signaling pathways lead to rapid PI3K phosphorylation. PI3K is involved in LPS-mediated NF-κB activation [70]. Moreover, NF-κB can promote the expression of several pro-inflammatory cytokines and is inextricably linked to inflammation development [71]. Akt, a PI3K target, is involved in the inflammatory response [72]. LPS activates PI3K through TLR4 and leads to AKT activation. PI3K promotes the NF-κB transcriptional activity and TNF-α production, while PI3K inhibition decreases TNF-α production [36]. We validated the TLR4/PI3K/Akt/NF-κB signaling pathway, and IHC revealed that TLR4, Myd88, PI3K, Akt, and NF-κB p65 protein levels were significantly decreased in the pharyngeal tissues of CP rats in the treatment group when compared with that in the model group. These results suggest that QYF treatment could effectively exert a protective effect on the rat pharynx by downregulating TLR4/PI3K/Akt/NF-κB pathway-related proteins (Fig. **11**). In addition, we validated the TLR4/PI3K/Akt/NF-κB signaling pathway and found that the low-dose group showed better improvement compared to the high-dose group. This could be due to the loss of active substances during the concentration process in the high-dosage group [73-75]. Therefore, our future research will focus on investigating the impact of the QYF decoction and the concentration process on the drug's efficacy in SD rats.

## CONCLUSION

This network pharmacology study revealed the potential mechanisms for the multi-component, multi-target, and multi-pathway actions of QYF in CP treatment. By establishing an innovative anthropomorphic “spicy overeating” animal model, we found that the QYF reduced the serum LPS levels, inhibited the abnormal expression of the TLR4/PI3K/Akt/NF-κB signaling pathway, reduced the levels of TNF-α, IL-1β, and IL-6, and improved the foot and ear swelling in CP rats. These results suggested that QYF exerted its therapeutic effect on CP by inhibiting LPS production and regulation of the TLR4/PI3K/Akt/NF-κB signaling pathway-related protein expression, thereby enhancing the anti-inflammatory capacity *in vitro* and *in vivo*. However, whether other pathways are involved in QYF-mediated amelioration of CP caused by “excessive consumption of spicy food and alcohol” needs further validation. We intend to investigate other pathways and inflammatory factors in future studies.

## Figures and Tables

**Fig. (1):**
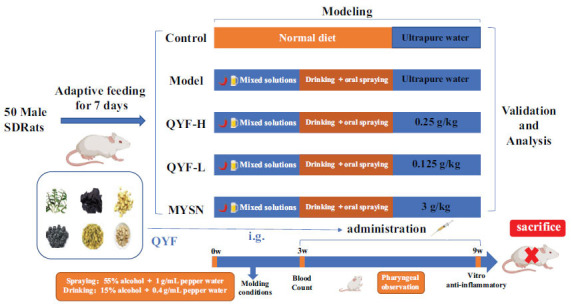
Flow chart for *in vivo* validation of animal experimental protocols. i.g., intragastric administration. *“Created with MedPeer (medpeer. cn)”.*

**Fig. (2):**
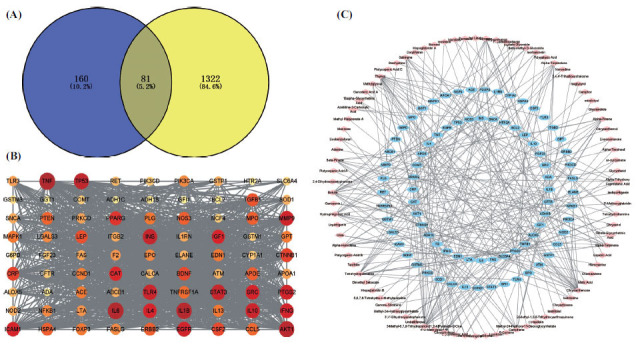
Network pharmacological analysis of QYF for CP. (**A**) Venn diagram depicting QYF drug-CP interacting genes. (**B**) Constructed maps of compound-target networks associated with drugs and CP. (**C**) The PPI network of a QYF drug for CP.

**Fig. (3):**
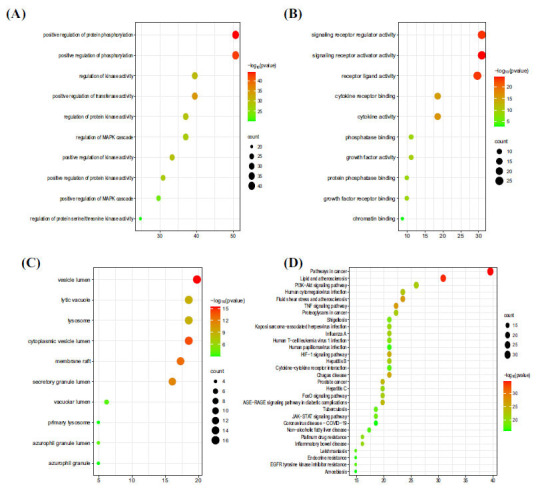
Functional enrichment of the target genes and screening of the core signaling pathway. (**A**) A bubble diagram depicting the BP analysis. (**B**) A bubble diagram depicting the MF analysis. (**C**) A bubble diagram depicting the MF analysis. (**D**) A bubble diagram depicting the KEGG analysis.

**Fig. (4):**
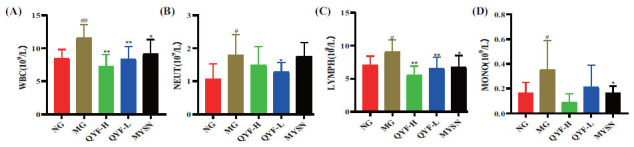
Effects of QYF treatment on the blood routine of rats. Changes in whole blood WBC (**A**), NEUT (**B**), LYMPH (**C**), and MONO (**D**) counts in rats after 6 weeks of drug administration. When compared with the normal control group, ^#^*P* < 0.01, ^#^*P* < 0.05; when compared with the model control group, **P < 0.01, *P < 0.05.

**Fig. (5):**
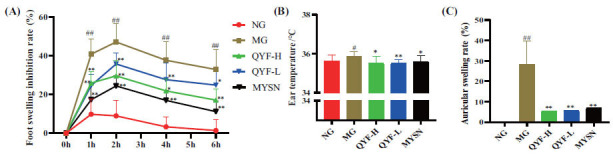
The results of QYF anti-inflammatory efficacy. The effect of QYF on foot swelling inhibition rate (**A**), ear temperature (**B**), and auricular swelling rate (**C**) in CP rats. When compared with the normal control group, ##*P* < 0.01, #*P* < 0.05; when compared with the model control group, **P < 0.01, *P < 0.05. When compared with the normal control group, ##*P* < 0.01, #*P* < 0.05; when compared with the model control group, **P < 0.01, *P < 0.05.

**Fig. (7):**
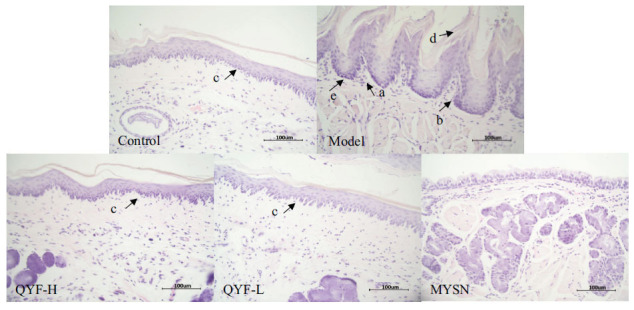
Effect of QYF on pharyngeal HE staining (SP× 400). (**A**) Inflammatory cells. (**B**) Mucosal epithelium. (**C**) Muscle fiber. (**D**) Slime. (**E**) Protruding nail.

**Fig. (8):**
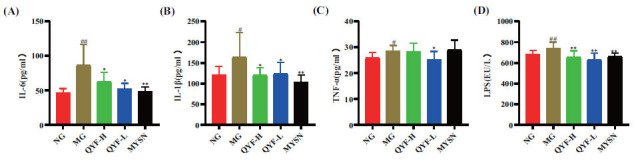
The effect of QYF on the levels of serum IL-6 (**A**), IL-1β (**B**), TNF-α (**C**), and LPS (**D**) in CP rats. When compared with the normal control group, ^#^*P* < 0.01, ^#^*P* < 0.05; when compared with the model control group, **P < 0.01, *P < 0.05.

**Fig. (9):**
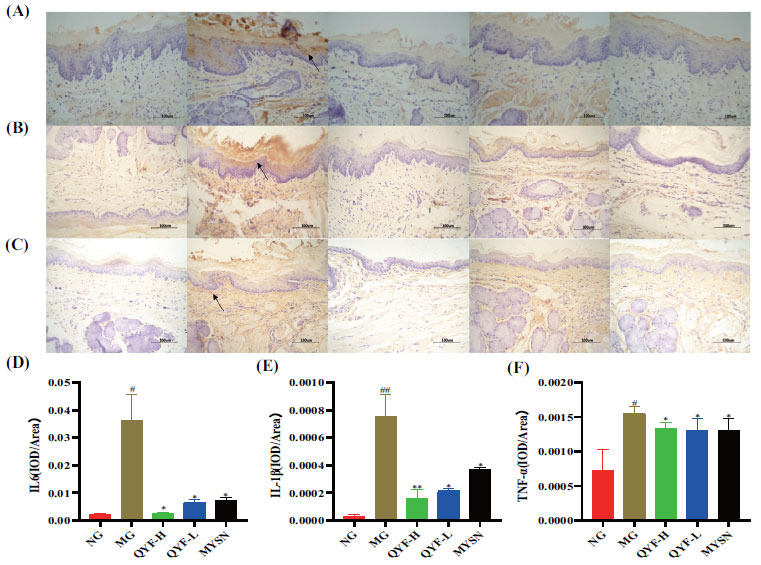
The effect of QYF on the levels of IL-6, IL-1β, and TNF-α inflammatory cytokines in the pharynx of CP rats. The expression level of relevant indicators is directly proportional to color, and the higher the expression level, the darker the tissue color. Representative micrographs (400×) of immunohistochemical staining of the rat pharynx for IL-6 (**A**), IL-1β (**B**), and TNF-α (**C**). Imagine a Pro-quantified rat pharynx for IL-6 (**D**), IL-1β (**E**), and TNF-α (**F**) IOD/area. When compared with the normal control group, ^#^*P* < 0.01, ^#^*P* < 0.05; when compared with the model control group, **P < 0.01, *P < 0.05.

**Fig. (10):**
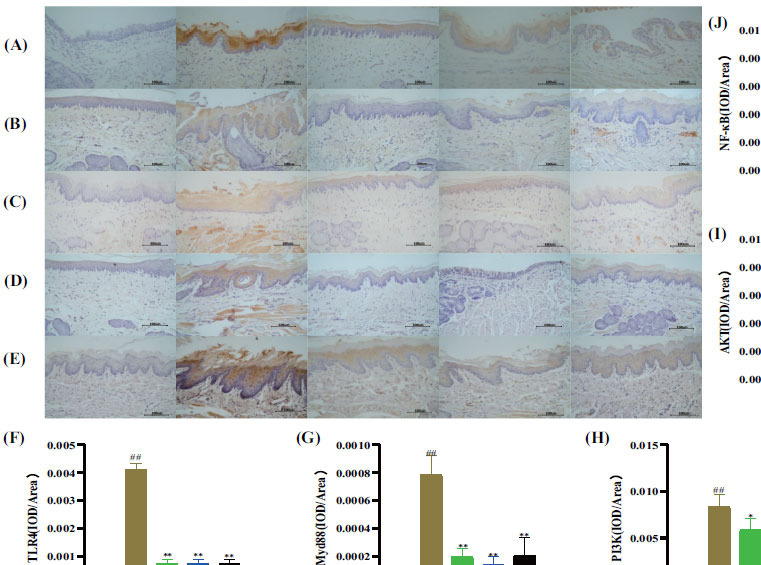
The effect of QYF on the TLR4/PI3K/Akt/NF-κB signaling pathway. The expression level of relevant indicators is directly proportional to color, and the higher the expression level, the darker the tissue color. The expressions of rat TLR4 (**A**), Myd88 (**B**), AKT (**C**), PI3K (**D**), and NF-κB p65 (**E**) were observed by IHC at 400x magnification. TLR4 (**F**), Myd88 (**G**), AKT (**H**), PI3K (**I**), and NF-κB p65 (**J**) proteins were quantified as the IOD/area by Imagine Pro. When compared with the normal control group, ^#^*P* < 0.01, ^#^*P* < 0.05; when compared with the model control group, **P < 0.01, *P < 0.05.

**Table 1:** Scoring criteria for throat tissue injury.

**Rating**	**Pharyngeal Phenotype**
0	The throat tissue was light red, with a moist and shiny surface, no secretions, no congestion, swelling and other pathological phenomena.
1	Poorly glossy throat tissue, little secretion, and little acute congestion.
2	The color of the throat was dark red, poorly colored, secreted, and accompanied by acute, mild swelling.
3	The throat tissue was dark red, with greatly increased mucus production, acute congestion, and marked swelling.

**Table 2:** Information on effective components of *Dendrobium officinale.*

**MOL ID**	**Active Ingredients**	**References**
MOL004328	Naringenin	[27]
MOL002322	Isovitexin	[28]
MOL005190	Eriodictyol	[29]
MOL003044	Chryseriol	[29]
MOL004576	Taxifolin	[29]
MOL000354	Isorhamnetin	[29]
MOL008647	Moupinamide	[30]
MOL000483	(Z)-3-(4-hydroxy-3-methoxy-phenyl)-N-[2-(4-hydroxyphenyl)ethyl] acrylamide	[27]
MOL000359	β-sitosterol	[31]
CID5315859	Chrysotoxene	BATMAN

**Table 3:** Active component information.

**Active Ingredients**	**Source**	**Active Ingredients**	**Source**
Azetidine-2-Carboxylic Acid	*Polygonatum cyrtonema*	Sinapic Acid	*Glycyrrhiza uralensis*
Aspartic Acid	*Polygonatum cyrtonema*	Licoricesaponine C2	*Glycyrrhiza uralensis*
Digitalis Glycoside	*Polygonatum cyrtonema*	Licorisoflavan A	*Glycyrrhiza uralensis*
Homoserine	*Polygonatum cyrtonema*	Isoramanone	*Glycyrrhiza uralensis*
Vitexin Xyloside	*Polygonatum cyrtonema*	Gancaonin F	*Glycyrrhiza uralensis*
Mannose	*Polygonatum cyrtonema*	Glycyrol	*Glycyrrhiza uralensis*
2,4-Dihydroxyacetophenone	*Chrysanthemi Flos*	Licoricesaponine A3	*Glycyrrhiza uralensis*
Chrysanthenone	*Chrysanthemi Flos*	3,3’-Dimethylquercetin	*Glycyrrhiza uralensis*
Borneol	*Chrysanthemi Flos*	Dimethyl Sebacate	*Glycyrrhiza uralensis*
Chlorochrymorin	*Chrysanthemi Flos*	Neoisoliquiritin	*Glycyrrhiza uralensis*
Tetradecanal	*Chrysanthemi Flos*	Ononin	*Glycyrrhiza uralensis*
Chrysanthetriol	*Chrysanthemi Flos*	Schaftoside	*Glycyrrhiza uralensis*
Cinaroside	*Chrysanthemi Flos*	Isotrilobine	*Glycyrrhiza uralensis*
Chamazulene	*Chrysanthemi Flos*	Licoricesaponine J2	*Glycyrrhiza uralensis*
Caryophyllene	*Chrysanthemi Flos*	Vicianin	*Glycyrrhiza uralensis*
Alpha-Pinene	*Chrysanthemi Flos*	Licoricesaponine A3	*Glycyrrhiza uralensis*
Tricosane	*Chrysanthemi Flos*	Narwedine	*Glycyrrhiza uralensis*
Beta-Pinene	*Chrysanthemi Flos*	Tetrahydroharmine	*Glycyrrhiza uralensis*
Camphene	*Chrysanthemi Flos*	Glycyrrhiza-Flavonol A	*Glycyrrhiza uralensis*
P-Cymene	*Chrysanthemi Flos*	Gancaonin A	*Glycyrrhiza uralensis*
Stachydrine	*Chrysanthemi Flos*	2-Methyl-1,3,6-Trihydroxyanthraquinone	*Glycyrrhiza uralensis*
Chrysanthemol	*Chrysanthemi Flos*	Licopyranocoumarin	*Glycyrrhiza uralensis*
Camphor	*Chrysanthemi Flos*	Uralenol	*Glycyrrhiza uralensis*
Lonicerin	*Chrysanthemi Flos*	Lupiwighteone	*Glycyrrhiza uralensis*
Alpha-Terpinolene	*Chrysanthemi Flos*	Glyzaglabrin	*Glycyrrhiza uralensis*
Alpha-Humulene	*Chrysanthemi Flos*	Glycyrrhetol	*Glycyrrhiza uralensis*
Adenine	*Chrysanthemi Flos*	Gancaonin D	*Glycyrrhiza uralensis*
Chrysanthemin	*Chrysanthemi Flos*	Glycyrrhizic Acid	*Glycyrrhiza uralensis*
Aminozide	*Chrysanthemi Flos*	2,4,4’-Trihydroxychalcone	*Glycyrrhiza uralensis*
Sabinene	*Chrysanthemi Flos*	Ononitol	*Glycyrrhiza uralensis*
Tilianin	*Chrysanthemi Flos*	Licofuranocoumarin	*Glycyrrhiza uralensis*
1,8-Cineole	*Chrysanthemi Flos*	3’-Methoxyglabridin	*Glycyrrhiza uralensis*
Farnesol	*Chrysanthemi Flos*	Alpha-Trihydroxy Coprostanic Acid	*Glycyrrhiza uralensis*
26-Chloro-26-Deoxycryptogenin	*Chrysanthemi Flos*	Gancaonin P-3’-Methylether	*Glycyrrhiza uralensis*
Nerolidol	*Chrysanthemi Flos*	Licoricidin	*Glycyrrhiza uralensis*
Heneicosane	*Chrysanthemi Flos*	Glisoflavanone	*Glycyrrhiza uralensis*
3',4'-Dihydroxyacetophenone	*Chrysanthemi Flos*	Licochalcone A	*Glycyrrhiza uralensis*
Alpha-Terpineol	*Chrysanthemi Flos*	3,3'-Dimethylquercetin	*Glycyrrhiza uralensis*
Luteolin	*Chrysanthemi Flos*	Narcissin	*Glycyrrhiza uralensis*
Myrcene	*Chrysanthemi Flos*	Hispaglabridin B	*Glycyrrhiza uralensis*
Acacetin	*Chrysanthemi Flos*	Isolicoflavonol	*Glycyrrhiza uralensis*
Thymol	*Chrysanthemi Flos*	8-Methoxy-5-O-Glucoside Flavone	*Glycyrrhiza uralensis*
Naringenin-4'-Glucoside-7-Neohesperidoside	*Mume Fructus*	Isoquercitrin	*Glycyrrhiza uralensis*
Naringenin	*Mume Fructus*	Isoononin	*Glycyrrhiza uralensis*
Amygdalin	*Mume Fructus*	Licoricone	*Glycyrrhiza uralensis*
Oleanolic Acid	*Mume Fructus*	Phebalosin	*Glycyrrhiza uralensis*
Prunasin	*Mume Fructus*	Urea	*Glycyrrhiza uralensis*
Citric Acid	*Mume Fructus*	Neouralenol	*Glycyrrhiza uralensis*
Hydroginkgolinic Acid	*Mume Fructus*	Glyyunnanprosapogenin D	*Glycyrrhiza uralensis*
Malic Acid	*Mume Fructus*	3-Hydroxyglabrol	*Glycyrrhiza uralensis*
Tartaric Acid	*Mume Fructus*	Neowilforine	*Glycyrrhiza uralensis*
Methyl Montanate	*Platycodon grandiflorum*	Licoflavone	*Glycyrrhiza uralensis*
Platycogenic Acid B	*Platycodon grandiflorum*	Uralenneoside	*Glycyrrhiza uralensis*
Platycodin A	*Platycodon grandiflorum*	Glycyrrhizin	*Glycyrrhiza uralensis*
Methyl Platyconate A	*Platycodon grandiflorum*	Licobenzofuran	*Glycyrrhiza uralensis*
Platycogenic Acid A	*Platycodon grandiflorum*	Isoorientin	*Glycyrrhiza uralensis*
Polygalacin D2	*Platycodon grandiflorum*	3,4-Dicaffeoyl-5-(3-Hydroxy-3-Methyl) Glutaroyl Quinic Acid	*Glycyrrhiza uralensis*
Platycogenic Acid C	*Platycodon grandiflorum*	Formononetin	*Glycyrrhiza uralensis*
Betulin	*Platycodon grandiflorum*	Isogosferol	*Glycyrrhiza uralensis*
Platycodin C	*Platycodon grandiflorum*	Licoricesaponine G2	*Glycyrrhiza uralensis*
Platyphylline	*Platycodon grandiflorum*	Isoliquiritin	*Glycyrrhiza uralensis*
Beta-Methyl-D-Glucoside	*Platycodon grandiflorum*	Licoricesaponine D3	*Glycyrrhiza uralensis*
Platycodin D	*Platycodon grandiflorum*	Phaseollinisoflavan	*Glycyrrhiza uralensis*
Spinoside A	*Platycodon grandiflorum*	Ferulic Acid	*Glycyrrhiza uralensis*
Platycodigenin	*Platycodon grandiflorum*	Licoricesaponin C2	*Glycyrrhiza uralensis*
Polygalacin D	*Platycodon grandiflorum*	Ethyl-N-Buthy-Uralsaponin A Esters	*Glycyrrhiza uralensis*
Polygalacic Acid	*Platycodon grandiflorum*	Gancaonin B	*Glycyrrhiza uralensis*
N-Methylplatydesmin	*Platycodon grandiflorum*	Methyl-24-Hydroxyglycyrrhetate	*Glycyrrhiza uralensis*
Alpha-Spinasterol-Beta-D-Glucoside	*Platycodon grandiflorum*	Licoleafol	*Glycyrrhiza uralensis*
18beta-Glycyrrhetinic Acid	*Glycyrrhiza uralensis*	Glyuranolide	*Glycyrrhiza uralensis*
Glycyrrhetinic Acid	*Glycyrrhiza uralensis*	Glyeurysaponin	*Glycyrrhiza uralensis*
Ruvoside	*Glycyrrhiza uralensis*	5,6,7,8-Tetrahydro-4-Methylquinoline	*Glycyrrhiza uralensis*
Nicotiflorin	*Glycyrrhiza uralensis*	Gancaonin E	*Glycyrrhiza uralensis*
Corylifolinin	*Glycyrrhiza uralensis*	Licocoumarone	*Glycyrrhiza uralensis*
Licoricesaponine F3	*Glycyrrhiza uralensis*	Isoliquiritigenin	*Glycyrrhiza uralensis*
18alpha-Glycyrrhetinic Acid	*Glycyrrhiza uralensis*	Methyl 2-Hydroxy-3,4-Dimethoxy Benzoate	*Glycyrrhiza uralensis*
Gancaonin C	*Glycyrrhiza uralensis*	Neoliquiritin	*Glycyrrhiza uralensis*
Methyl Linoleate	*Glycyrrhiza uralensis*	Ganoderic Acid A	*Glycyrrhiza uralensis*
4'-O-Methylglabridin	*Glycyrrhiza uralensis*	Sigmoidin B	*Glycyrrhiza uralensis*
Isoschaftoside	*Glycyrrhiza uralensis*	Uralene	*Glycyrrhiza uralensis*
Monoammonium Glycyrrhizinate	*Glycyrrhiza uralensis*	Uralsaponin A	*Glycyrrhiza uralensis*
Isoliensinine	*Glycyrrhiza uralensis*	N-Tricosane	*Glycyrrhiza uralensis*
Dimethyl Sebacate	*Glycyrrhiza uralensis*	Glycyroside	*Glycyrrhiza uralensis*
Neoisoliquiritin	*Glycyrrhiza uralensis*	Licoricesaponine K2	*Glycyrrhiza uralensis*
Ononin	*Glycyrrhiza uralensis*	Uralsaponin B	*Glycyrrhiza uralensis*
Schaftoside	*Glycyrrhiza uralensis*	Umbelliferone	*Glycyrrhiza uralensis*
Isotrilobine	*Glycyrrhiza uralensis*	Astragalin	*Glycyrrhiza uralensis*
Licoricesaponine J2	*Glycyrrhiza uralensis*	Glycyrrhisoflavone	*Glycyrrhiza uralensis*
Vicianin	*Glycyrrhiza uralensis*	Gamma-Sitosterol	*Glycyrrhiza uralensis*
2,5-Dihydroxymethyl-3,4-Dihydroxypyrrolidine	*Glycyrrhiza uralensis*	Glycycoumarin	*Glycyrrhiza uralensis*
Gloeosteretriol	*Glycyrrhiza uralensis*	Uralenol-3-Methylether	*Glycyrrhiza uralensis*
Glycyrin	*Glycyrrhiza uralensis*	Liquiritin	*Glycyrrhiza uralensis*
Uralenin	*Glycyrrhiza uralensis*	Liquoric Acid	*Glycyrrhiza uralensis*
Neohancoside A	*Glycyrrhiza uralensis*	Gmelofuran	*Glycyrrhiza uralensis*
Liquiritigenin-7,4'-Diglucoside	*Glycyrrhiza uralensis*	Methyl-24-Hydroxy-11-Deoxoglycyrrhetate	*Glycyrrhiza uralensis*
Gancaonin I	*Glycyrrhiza uralensis*	Hispidulin	*Glycyrrhiza uralensis*
Glycyphyllin	*Glycyrrhiza uralensis*	Isotrifoliol	*Glycyrrhiza uralensis*
8-Methyl-10-Hydroxylycoctonine	*Glycyrrhiza uralensis*	Liquiritigenin	*Glycyrrhiza uralensis*
Rutin	*Glycyrrhiza uralensis*	Glycyrrhisoflavanone	*Glycyrrhiza uralensis*
Methylglyoxal	*Glycyrrhiza uralensis*	3-Methyl-6,7,8-Trihydropyrrolo [1,2-a]Pyrimidin-2-One	*Glycyrrhiza uralensis*
Hispaglabridin A	*Glycyrrhiza uralensis*	Tetrahydropalmatine	*Glycyrrhiza uralensis*
Isoglycyrol	*Glycyrrhiza uralensis*	*-*	-

**Table 4:** The active constituents of QYF regulate the target information of CP.

**Disease Name**	**Number of Targets**	**Target Name**
CP	81	TP53, TNF, NCF4, IL-6, ATM, IL-10, IFNG, IL-1β, CRP, TGFB1, CFTR, STAT3, ACE, CCND1, IL-4, BCL2, ELANE, IL-13, CSF2, PTGS2, TLR4, EDN1, MMP9, ADA, ABCB1, GFI1, NFKB1, GPT,I NS, GSTM1, MPO, COMT, EPO, TNFRSF1A, FAS, ADH1C, CCL5, LEP, BDNF, SOD1, ICAM1, NOS3, NOD2, EGFR, PTEN, F2, IL1RN, HTR2A, AKT1, LTA, FOXP3, ADH1β, GSTP1, FGF23, APOE, IGF1, PIK3CA, APOA1, ALOX5, FASLG, MAPK1, LGALS3, TLR3, GSTM3, PRKCD, G6PD, ERBB2, HSPA4, PIK3CD, CALCA, PLG, CTNNB1, CYP1A1, SNCA, CAT, SLC6A4, ITGB2, RET, SRC, GGT1, PPARG

## Data Availability

The data used to support the findings of this study are available from the researchers upon request.

## LIST OF ABBREVIATIONS

BP: Biological Process
CC: Cellular Component
CP: Chronic Pharyngitis
DL: Drug-Likeness
DL: Drug-like Property
ENT: Ear, Nose, and Throat
MF: Molecular Function
OB: Oral Bioavailability
PPI: Protein-protein Interaction
QYF: *D. officinale* Throat-clearing Formula
TCM: Traditional Chinese Medicine
TCMSP: Traditional Chinese Medicine Systems Pharmacology Database and Analysis Platform
